# Masculinity and Help-Seeking Among Men With Depression: A Qualitative Study

**DOI:** 10.3389/fpsyt.2020.599039

**Published:** 2020-11-24

**Authors:** Tobias Staiger, Maja Stiawa, Annabel Sandra Mueller-Stierlin, Reinhold Kilian, Petra Beschoner, Harald Gündel, Thomas Becker, Karel Frasch, Maria Panzirsch, Max Schmauß, Silvia Krumm

**Affiliations:** ^1^Department of Psychiatry II, University of Ulm and District Hospital Günzburg, Ulm, Germany; ^2^Department of Psychosomatic Medicine and Psychotherapy, University Hospital Ulm, Ulm, Germany; ^3^Department of Psychiatry, Psychotherapy and Psychosomatics, District Hospital Donauwörth, Donauwörth, Germany; ^4^Department of Psychiatry, Psychotherapy and Psychosomatics, University of Augsburg and District Hospital Augsburg, Augsburg, Germany

**Keywords:** masculinity, depression, help-seeking, service use, qualitative study

## Abstract

**Background:** Many studies indicate that men are more reluctant to seek help for mental health problems than women. Traditional ideas of masculinity are often seen as a cause of this phenomenon. However, little is known about the diversity of experiences during the processes of help-seeking and service use among men with depression who have already utilized mental health services. This study aims to explore men's experiences and attitudes toward depression, help-seeking, and service use in order to develop gender-sensitive services.

**Methods:** Narrative-biographical interviews were conducted with men treated for depression (*n* = 12). Interview topics included individual experience with depression, help-seeking behavior, and mental health service use. Transcripts were analyzed using qualitative content analysis.

**Results:** Before seeking treatment, men's help-seeking behavior was negatively affected by internalized masculine norms. However, findings indicate a change of attitudes toward depression after mental health service use. Men with depression emphasized a salutogenic perspective toward mental health problems and critically reflected on masculine norms. The positive function of men-only groups were described as key for successful service use.

**Conclusions:** Men with depression reported experiences toward help-seeking and service use on four different levels: (i) attitudes toward depression, (ii) perception of societal views on depression, (iii) experiences within the family context and (iv) experiences with mental health services. Interventions to reduce the stigma of being “unmanly” and to improve men's capacity to cope with being unable to work should be developed. Peer-led men-only groups may increase participants' self-esteem and assist in disclosing weaknesses. In the context of GPs' mediating role, training for health professionals concerning the impact of masculine norms on mental health is recommended.

## Introduction

There is broad evidence of men's reluctance to seek help for mental health problems. Studies support the generally-held assumption that men are less likely than women to get assistance from mental health professionals for problems (1). A body of empirical research has explored reasons for help-seeking decisions as well as service use behavior among men with depression. Research often suggests that men's help-seeking behavior regarding depression is attributed to traditional masculine norms such as being strong, successful, self-reliant, in control, and capable, along with an emphasis on avoiding emotions (2). In line with this, having depression is described as being “incompatible” with traditional masculinity due to the fact that emotional experiences in depression are linked to femininity; depression is often accompanied by feelings of powerlessness and lack of control; and the experience of depression often leaves people feeling weak and vulnerable (3). Results of a systematic review of qualitative studies on men's views of depression confirmed the impact of norms concerning masculine roles on men's attitudes toward depression and help-seeking (4). Receiving support or seeking help was associated with the risk of being ridiculed or marginalized as well as being seen as “unmanly” by others.

Further studies of a systematic review refer to the adverse effects of male role expectations and social pressures to perform well as family providers and fathers with consequences for help-seeking behavior. Depression was frequently perceived as a threat to men's roles as family provider and many participants reported feelings of inadequacy and incapability compared to their situation before depression (4). Compared to other severe illnesses, depression was described as a “particular challenge to masculinity” and experienced as “otherness” either in regard to other men or compared to the person they used to be (4). Fathers with mental health problems experienced hospitalization and medication as a disruption to their lives and the lives of their family members and prevented them from “being there” for their partners (5). A meta-analytic review on effects of paternal depression on fathers' parenting behaviors supports this assumption. Studies indicated that paternal depression has significant, though small, effects on parenting, with depressed fathers demonstrating decreased positive and increased negative parenting behaviors (i.e., parental engagement) (6). In contrast to the adverse effects of family role expectations, research highlighted the supportive function of the family during the help-seeking process (7, 8).

In addition to the impact of expectations regarding male roles on help-seeking behavior, studies revealed positive as well as negative experiences of (mental) health service use among men with depression. A recent qualitative study referred to conflicts that men experienced in relation to antidepressant use (9). On the one hand, medication was perceived as a way in which men asserted their control over difficulties; on the other hand, antidepressants were seen as an obstacle to emotional and physical vitality, for example by undermining sexual function. Further findings referred to the role of general practitioners (GPs) in the context of treating men's depression. Although studies pointed out that men were repeatedly found to be half as likely to seek help for mental health concerns from a GP compared to women (7), others emphasized GPs' function as a pathway to mental health services (10). Further studies revealed ways men communicate their depression within in- and outpatient services. While discussions about depression with health care providers were described as atypical for men (11), studies explored whether a change of setting improved access to treatment for common mental disorders in the context of mental health services. Research found that a higher proportion of men with mental health problems sought help via psychotherapeutic consultation in the workplace compared to standard psychosomatic outpatient care (12).

Even though traditional masculine norms play an important role in reinforcing men's reluctance to seek help, qualitative studies showed that some men seemed to benefit from just the same norms by perceiving these ideals as a healthy resource (13). While some men associated depression with powerlessness and lack of control, others described the recovery as a heroic struggle from which they emerged much stronger (3). Furthermore, there is some evidence that men do not necessarily subscribe to traditional ideals but demonstrate alternative forms of masculinity (4). These studies suggested that some men dissociated themselves from traditional masculine norms by emphasizing their sensitivity in coping with and utilizing in- and outpatient services due to depressive symptoms (3).

Despite emerging evidence for the diversity of men's experiences of help-seeking and service use, many studies provide a one-dimensional understanding of mental health behavior among men with depression, including reduced service use. Beyond this, there is a lack of knowledge on how men's specific needs in cases of depression are addressed by mental health services. Moreover, the impact of norms concerning traditional masculine roles for men with depression who have already utilized mental health services is unclear. Previous studies on mental health professionals' view about the impact of male gender for the treatment of men with depression stress the need to develop gender-sensitive services (14). On the one hand, results refer to the need of awareness of the role of gender and that its implications for mental health treatment should be an integral part of mental health professionals' education and the everyday practice of mental health treatment. On the other hand, more evidence is needed to develop mental health services based on the experiences of men with depression. This study therefore aims to explore experiences and attitudes toward depression, help-seeking and service use among men with depression who have already utilized mental health services in order to develop gender-sensitive services.

## Materials and Methods

This qualitative investigation is part of the mixed-methods study “Constructions of Masculinity and Mental Health Behavior of Men with Depression” (MenDe) funded by the German Research Foundation. The study aims for a comprehensive analysis of men's constructions of masculinity and the consequences for their mental health behavior. Through an analysis of the diversity of concepts of masculinity, the study contributes to a more detailed picture of depression among men.

### Selection of Participants for Qualitative Interviews

In the first step, based on a sample of 250 men with depression, a latent class analysis was performed and three types of a combination of masculinity orientation and job-related attitudes were identified (15). In the second step, twelve biographical interviews with four representatives of each class were conducted in order to get a deeper understanding of class membership in respect to subjective illness theories and coping processes among men with depression (16). In a third step, these interviews were re-analyzed in order to gain a deeper understanding of men's subjective perspectives on help-seeking barriers and facilitators of service use irrespective of class membership. This article focuses on this third step by addressing men's perspectives on help-seeking decisions and service use experiences.

### Recruitment

Participants were recruited both inside and outside healthcare settings in Southern Germany. Eligible patients were asked by doctors or other clinical staff about their willingness to participate in the study. After expressing their willingness to participate, research workers contacted the patients, informed them about the aims of the study and verified the patients according to the following inclusion criteria: patients must be male, aged between 18 and 64, diagnosed or self-identified as having depression and sufficient German language skills. The study's exclusion criteria were organic mental disorder, dementia, anorexia with body mass index (BMI) <17, addiction and bipolar disorder or schizophrenia.

### Instrument

We conducted narrative-biographical interviews (Table 1) in which interviewees were given as much time as required to talk about their experiences during the help-seeking and service use process in their own words (17). Participants were encouraged to talk about their experience of depression, help-seeking behavior, and service use. In an additional part of the interview, we used a semistructured interview guide, which included the topics of illness theories, the social consequences of depression and personal coping strategies. At the end of the interviews, respondents were asked to describe whether and how masculine norms influenced their help-seeking decisions and service use experiences. One pilot interview was conducted with no changes for the interview guide.

**Table 1:** Interview guide.

**Topic**	**Question**
**Biographical interview section**	
Living with depression	As you know, we are especially interested in men's experiences with depression. We would like to know how it impacts your life, what are your coping strategies and what kind of services are helpful for you.
	We are not only interested in the acute phase of your depression, we would also like to ask about your whole life, i.e., how the depression developed. Could you describe step by step from past to present what your experiences were?
Experiencing first symptoms of depression until…	…You told me, that you went to a GP/into a clinic (or similar). Could you tell me what led to that visit and what happened next?
Receiving the diagnosis depression until…	…You told me, that you went into a clinic (or similar). Did you receive a diagnosis? Could you elaborate on how the visit/stay went?
Mental service use until…	…you told me, that you received treatment for depression. Could you tell me about your treatment in depth? What kind of experiences did you have in the clinic (or similar)?
**Semistructured interview section**	
Evaluation of mental health services	How do you review and rate the mental health services, retrospectively?
Illness theory	What caused your depression in your opinion?
Life change	How has illness changed your life (your person, others, professionally, and privately)?
Coping with depression	What helps you to cope with your illness?
	What makes it difficult?
Masculinity and depression	Could you explain whether and if yes how masculine norms influenced your help-seeking decisions and service use experiences?
Undiscussed themes	Is there anything, that was not discussed during this interview but is important to you?

### Data Collection

Twelve interviews were conducted by a researcher (TS) between March and June 2018. The place for the interviews was chosen by the respondents. Interviews took place either at home or in the facilities of Ulm University, from which audio was recorded, transcribed verbatim, and anonymized. The duration of the interviews was between 27 and 133 min, with a mean of 75 min. Respondents were asked to answer socio-demographic questions at the end of the interview.

### Analysis

Interview transcripts were analyzed using qualitative content analysis (18) via the following steps: (i) potential categories were defined, derived deductively from the research question and theoretical background (e.g., mental health service use); (ii) inductive codes were formulated based on the material (e.g., experienced stigma); (iii) codes were collated into potential themes; these themes were checked for consistency with coded extracts across the dataset, and were refined and summarized into categories. The interviews were coded independently by three researchers (MSt, SK, TS) so that coding could be compared. Discordant coding was discussed in a qualitative research group until consensus was reached. It should be reflected that the interviewer was a male researcher. Against the background of interactionism, male participants might answer in a specific way depending on the gender of the interviewer (for e.g., to stage themselves as “real men” who never lost control in coping depression). To try to control this bias a qualitative research group with several perspectives (men and women, different professions etc.) discussed this issue critically. We used MAXQDA 12 for data analysis.

### Characteristics of Participants

Interviewees' characteristics (*n* = 12) are presented in Table 2. The mean age was 52 (range from 30 to 62). Three participants had a general qualification for university entrance, five an advanced technical college entrance qualification, three an intermediate school-leaving certificate, and one participant had a certificate of secondary education. The mean household income was 3,917€ (range from 2,500€ to 6,000€). Interviewees were employed in technical professions, public administration, marketing, social profession, or transportation. Five participants were unemployed, on sick leave, or participating in occupational rehabilitation. Before being on sick leave, in rehabilitation or unemployed these interviewees were employed in manufacturing and construction, human services, and marketing. One participant was single, two were divorced and nine were married. Seven participants had children and two lived together with them. At the time of the interviews, eleven out of twelve participants were utilizing mental health services by a GP, psychologist and/or a psychiatrist.

**Table 2:** Characteristics of the clinical sample of participants (*n* = 12).

Name^*^	**Age range**	**Occupational field (originally field of work)**	**Marital status**	**Children/Number**	**Living together with children**	**Current treatment**
Steve	50–54	Social profession	Divorced	3	No	Psychologist Psychiatrist GP
James	55–59	On sick leave (construction)	Married	–	–	Psychologist
Daniel	50–54	Unemployed	Married	2	No	Psychologist Psychiatrist GP
George	45–49	Rehabilitation (construction)	Married	1	Yes	Psychologist Psychiatrist
Jack	50–54	On sick leave (marketing)	Married	–	–	Psychologist Psychiatrist GP
Oliver	55–59	Public administration	Married	3	Yes	Psychologist
Luke	55–59	Technical profession	Married	2	No	Psychologist
Mark	50–54	Marketing	Divorced	–	–	Psychiatrist
Alex	55–59	Transportation	Married	2	No	None
Edward	60–64	On sick leave (human services)	Married	2	No	Psychologist
Henry	30–34	Technical profession	Single	–	–	Psychologist
Harry	45–49	Technical profession	Married	–	–	Psychiatrist

* *Pseudonym*.

## Results

Based on the qualitative analysis, we summarized themes which refer to men's (i) attitudes toward depression; (ii) perception of societal views on depression; (iii) Family environment: between role expectations and social support; and (iv) experiences with mental health services (Table 3). These main themes contained 20 categories with 58 subcategories and will be presented in more detail in the following section, using pseudonyms and participant's age range to preserve confidentiality.

**Table 3:** Core themes, categories, and subcategories, *n* = number of quotations.

**Core themes**	**Categories**	**Subcategories**	***n***
Men's attitudes toward coping with depression: critical stance toward masculine norms	Trivialized symptoms of depression	Temporary condition, normal state of health, downplaying symptoms	10
	Individualized problem solving	Own problems, refused help-seeking	7
	Hid depression due to masculine norms	Avoiding feelings, appearing strong, never crying, breadwinner	7
	Avoided help-seeking to save career options	Secrecy, maintaining employability, safeguard career option	6
	Change in attitudes toward mental health problems	wake-up call, changed harmful attitudes, self-competence, noticing warning signs earlier	11
	Salutogenic perspective on depression and help-seeking	depression as an important life experience, Chance to reflect attitudes, positive coping strategies, being sensitive	10
Men's perception of societal views on depression: the stigma of being depressed and “unmanly”	Assessed as being incapable of coping with distress	Not taken seriously by others, incompetent	12
	Stigma toward help-seeking and having depression	Clinic-related stigma, can't hide it, rejecting inpatient services	12
	Stigma toward the inability to work	Failure to fulfill norms, shunned by workmates, discrimination, lazy	10
	Stigma of being unmanly	Weak, vulnerable, looser	8
	Growing acceptance	Societal acceptance, become more open	8
Family environment: between role expectations and social support	Loss of empathy in the family environment related to mental health problems	Cannot mention depressive symptoms, annoying	14
	Depression not taken seriously	Pseudo-problem, trivialize	9
	Lack of understanding related to failure to recover	“inability” to recover, must be healthy	6
	Suffering from paternal role expectations	never recognized, fatherhood and role expectations	8
(Mental) health service use: between obstacles and enablers	Open-minded and appreciative environment as a coping resource	Lack of interest, downplaying depression, relativize depression	11
	Partners' emotional support	Supportive, encouraging, friendly contact	21
	Service users' social support during and after inpatient services	Face-to-face exchange, similar illness-related background	14
	Group counseling for men to facilitate disclosure of weaknesses	Feeling accepted, no questions why, listening, sympathy, and empathy	15
	Familiarity of peer-led men-only groups	Same gender, same problems, address anxieties, open up to someone	11

### Men's Attitudes Toward Coping With Depression: Critical Stance Toward Masculine Norms

The majority of interviewees reported that masculine norms influenced their attitudes toward depression as well as their decision to seek help. Some of them reported having “trivialized” their symptoms in terms of a “temporary” condition, which was expected to return quickly to a normal state of health: “It's a bit difficult at the moment, but it'll be fine again soon” (Luke, 55–59 y). Most participants reported having tried to solve mental health problems on their own instead of seeking mental health services. Along these lines, some interviewees described their own as well as men's socialization in general as having an emphasis on avoiding feelings, appearing strong and never crying. One participant reflected masculine norms meant that he never disclosed mental health problems, saying that “[…] it doesn't exist among men. Men are the breadwinners, the problem solvers, the doers” (Luke, 55–59 y). In order to meet traditional masculine norms concerning societal roles, respondents decided not to disclose their mental health problems and to post-pone their own needs:

You have to play your part in the world of business. This means you can rarely be honest anywhere. That's the main thing, not to actually show how you're really doing (Steve, 50–54 y).

Interviewees explained low levels of help-seeking behavior as being a means of maintaining their employability as well as to safeguard career options. Therefore, one respondent reported that shortly after being admitted to a psychiatric ward due to a mental breakdown, he asked to be discharged “in order to go to work” (Jack, 50–54 y). In line with a critical stance toward masculine norms, the analysis indicates a change in attitudes toward mental health problems during recovery processes. Interviewees emphasized a salutogenic perspective on depression and help-seeking. Respondents perceived having depression as an important “life experience” (Alex, 55–59 y) or a “necessary wake-up call” (Harry, 45–49 y). This perspective awakened them to the need to change harmful attitudes toward work and life: “Before my illness, work came first. And now I have to say I'm the top priority and I only do what is good for me” (Luke, 55–59 y). Some participants viewed their depression primarily as a chance to reflect on their attitudes, which led to positive coping strategies in everyday life (Alex, 55–59 y). Due to their critical stances toward masculine norms, interviewees recommended being “more sensitive to looking after oneself and noticing these warning signs earlier” (Luke, 55–59 y). Others suggested detaching oneself from traditional masculine norms that inhibit help-seeking and service use for depression: “This kind of thing, of ‘I cannot show weakness, I cannot be sick’ should be avoided” (Jack, 50–54 y).

### Men's Perception of Societal Views on Depression: The Stigma of Being Depressed and “Unmanly”

Participants referred to a variety of gender-related stigma experiences which could be classified into two categories: firstly, participants reported being assessed as incapable to adequately cope with mental distress. Depressive symptoms were not taken seriously by others who alleged that mental disorders are a result of an inability to deal with distress: “I've often heard people say ‘Get a grip! Don't make such a fuss’” (Luke, 55–59 y). Secondly, stigma experiences were related to the failure to fulfill norms relating to work. One respondent reported that he had been shunned by workmates and management due to his failure to cope with mental health problems: “They said I didn't appear to be sick and that they had never been sick in their lives” (Luke, 55–59 y). The analysis revealed that “not being sick” was associated with attributes of being strong, successful and self-reliant conveyed by the employment environment. In contrast, mental health problems in the workplace left interviewees feeling weak and vulnerable. Thus, they reported that their depression-related incapacity to work made them feel stigmatized by other colleagues. Participants were labeled “loser,” “lazy,” or “incapable” (James, 55–59 y). Along with these experiences, respondents on sick leave reported being afraid that “outside my house somebody could ask me ‘What are you doing for work these days?’” (Jack, 50–54 y) Some participants developed strategies in order to meet work-related norms, e.g., by telling people “I'm a freelancer. I'm working from home at the moment” (Jack, 50–54 y). Against this background, respondents stated that seeking help continues to be viewed negatively as it is connected with the inability to cope with mental distress: “It's certainly still the case that people say, ‘Oh, he needed help, he can't do it himself’” (Oliver, 55–59 y). Interviewees perceived little understanding of what it means to have a depressive disorder and seek help within different social and job-related contexts. Some respondents reported fears of being stigmatized, which led to them rejecting inpatient services:

The goal is under no circumstances to check into a clinic, because then the stigma is even bigger. That means you can't hide it any more, either at work or in your private life (Oliver, 55–59 y).

However, some participants perceived a slowly growing societal acceptance for professional help-seeking. One respondent noted that “People used to be locked up. All psychiatric institutions used to be completely closed off, and in the last 15 years they've become much more open” (Daniel, 50–54 y).

### Family Environment: Between Role Expectations and Social Support

Participants reported both negative and positive experiences within their familial context during the help-seeking process. Some men perceived a loss of empathy that might be related to the duration of mental health problems: “I feel like I can't really mention my depressive symptoms at home anymore, because obviously it's annoying [for my family]” (Oliver, 55–59 y). Participants described a lack of understanding regarding their depression and their “inability” to recover:

The worst thing is my environment: “You've been to the hospital twice now, you are taking the medication and you have been on holiday, you must be healthy now” (Jack, 50–54 y).

Others reported that the diagnosis of “depression” was not taken seriously by family members but seen as a pseudo-problem (Daniel, 50–54 y). Within the familial context, paternal role expectations were an important issue for some participants: “My family couldn't understand that I, a father, didn't go to the hardware store today, because I didn't feel good. It was never really recognized” (James, 55–59 y). In contrast, an open-minded and appreciative family environment was seen as assisting in the seeking of professional help (Harry, 45–49 y). Further findings underlined the supportive role of the partner as the “rock” (Jack, 50–54 y) in the help-seeking process: “Without my wife, I wouldn't still be sitting here. I wouldn't have accepted any help, and I would be sitting somewhere in a clinic where I wouldn't be able to open the door by myself” (Jack, 50–54 y).

### (Mental) Health Service Use: Between Obstacles and Enablers

Participants described both negative and positive experiences with (mental) health service use. Some respondents reported a lack of interest as well as a downplaying of depressive symptoms by GPs which led to them no longer seeking help:

The GP said, “Yeah, my God, I'm seeing you again? So, what have you got? Problems at work? So, a lot of people have problems. Don't get so upset!” (Oliver, 55–59 y).

In some men's views, GPs tended to relativize depressive symptoms and recommended calming oneself down. In contrast, other interviewees reported the role of GPs as being a gateway to mental health services and being generally supportive and encouraging as well: “I told him everything. And then he pressed a note into my hand and said I had to go to the clinic immediately” (Alex, 55–59 y). Alongside structures of formal service use, interviewees pointed out the positive role of other informal service users' social support during and after inpatient services. They reported the key role of a face-to-face exchange with fellow service users, especially those with a similar illness-related background, which was described as a supportive feature during the help-seeking process. Respondents felt accepted without being questioned by others: “There was no question of why… just this listening and sympathy and being there for you” (Luke, 55–59 y). In particular, group counseling for men with depression was perceived as facilitating the disclosure of weaknesses. For this reason, interviewees preferred approaches that enabled them to address anxieties in a group of fellow service users who identify with the same gender. Relatedly, participants highlighted the familiarity of men-only groups in the context of inpatient services: “You can really show your true self. You can show weakness and it won't be interpreted negatively. Nobody laughs at you” (Steve, 50–54 y). Because of societal expectations due to masculine norms as well as perceived stigma of being “unmanly,” some participants defined inpatient services as a sheltered space: “You're in a kind of cocoon, where you're protected, where you feel really comfortable. Where you're doing well” (Luke, 55–59 y). Others described inpatient services as being their first opportunity to open up to someone else: “That was the first time I was able to be open about my problems like that” (James, 55–59 y). These interviewees perceived inpatient services as being a protection against external expectations which they were unable to meet due to mental health problems. Instead of having to meet the expectation of being active and responsible, service users are allowed to be “passive” recipients:

I really appreciate the clinic. To be free of my responsibilities for a while. In a clinic, you're completely relieved of it. You're given a plan to work through (George, 45–49 y).

Consistent with the change in attitudes toward mental health-related help-seeking, interviewees described their experiences of inpatient services as an “educational resource”, where they could benefit from fellow patients' life experiences and learn how to cope with depressive symptoms: “I learned a lot through meeting people with the same problems. That makes you smarter, when you know how to deal with it” (Alex, 55–59 y).

## Discussion

The objective of our study was to explore experiences and attitudes toward depression, help-seeking and service use in a sample of men undergoing treatment for depression. Our findings suggest that men with depression retrospectively give both negative and positive experiences of help-seeking and service use. On the one hand, they report the adverse impact of masculine norms as well as stigma experiences. On the other hand, results indicate a transformation of their attitudes toward traditional masculine norms by critically reflecting on non-help-seeking behavior as well as maladaptive work patterns. In this regard, peer-led men-only groups were seen as assisting the disclosure of anxieties.

### Adverse Impact of Masculine Norms

Retrospectively, the interviewees perceived that they trivialized and downplayed their symptoms, which they justified using the societal role of caring for their family or to meet career-related expectations. This is in line with previous studies showing that men who suffer from depression have difficulties disclosing their mental health problems, reasoning that traditional masculine norms such as being strong, successful, and self-reliant inhibit help-seeking behavior (2). Furthermore, attitudes toward depression can be discussed following the concept of hegemonic masculinity (19). Hegemonic masculinity is defined as the dominant cultural ideal in Western countries serving as a normative orientation for men concerning heterosexuality, rationality, success, strength, or control, although only a small group of men might conform to these ideals (19). Therefore, the sociological concept describes a cultural ideal of masculinity in a given society, including being strongly work-oriented, having breadwinner mentality and a reluctance to talk about mental health issues. Results of qualitative studies support this assumption by showing that men's decision not to seek help is accompanied by the concern that they would be making fools of themselves and expect social isolation as a consequence of not matching masculine role norms (20).

### Male Stigma Surrounding Depression and Help-Seeking

Additionally, our findings also report on stigma experiences, which relate to the inability to cope with mental distress as well as to perform expected job- and family-related roles. The concept of mental illness stigma describes a process that involves labeling, stereotypes, separation, loss of status, and discrimination (21, 22). Two forms of stigma may be of relevance to our findings: public stigma, which involves processes that represent stereotypes, prejudice and discrimination among members of the general public (e.g., “all men with depression are weak and unmanly”) and self-stigmatization, where people with mental illness agree with negative stereotypes and turn them against themselves (e.g., “I am a man with depression and need help, so I must be weak”). Men with depressive symptoms may avoid treatment in order not to be labeled “mentally ill” or “unmanly” by others, and self-stigmatization or shame can undermine motivation to seek help (23). However, while much of the literature shows how masculinity creates stigma around men seeking help for depression, our findings may provide insight into how men were able to cope with stigma experiences by accessing (mental) health services. Analysis of our findings underlined the role of peer support, especially in men-only groups, which allowed men to disclose weaknesses without being questioned by others.

### GPs Role in Men's Help-Seeking Decisions

Some interviewees indicated negative experiences in seeking GPs' help for depression, whereas other participants pointed out the importance of GPs as gateways to mental health services. This discrepancy is in line with results from a qualitative study that identified positive factors that may assist men's help-seeking decisions (8). These findings suggest that men consulted a GP prior to counseling, which led to both negative and positive experiences. For some interviewees, the GP provided immediate assistance, while others reported the initial consultation as being a deterrent (8) to further help-seeking. Previous studies have identified a number of reasons for men's varying experiences when consulting a GP. Findings also referred to GPs' diagnostic errors in evaluating symptoms of depression in men, which can also lead to differing diagnoses (24). Others pointed out time restrictions that undermine GPs' capacity to effectively diagnose and treat depression (25). However, findings across different studies have been inconsistent, with previous research also finding that men emphasized GPs' supportive role and stressed that a positive relationship between patient and GP facilitates mental health service use (26). Therefore, more in-depth education of GPs in terms of handling psychosocial issues in their male patients in particular is recommended. Moreover, results indicate that some male GPs tended to play down symptoms of depression and primarily recommended reducing occupational stress making it more difficult for patients to seek help. In order to reduce this factor impairing the patients' ability to seek help, health professionals should be trained to reflect their own gender stereotypes, e.g., by participating in advanced gender trainings.

### Transformation of Attitudes During Recovery Processes

Contrary to the power of traditional masculine norms as an obstacle to seeking help, our findings indicate a change of attitudes toward service use during the participants' recovery processes. Our qualitative analysis pinpointed a critical stance toward masculine norms as well as a salutogenic perspective on depressive illness and service use experiences among interviewees after seeking help. In contrast to the assumption that psychiatric service use contradicts masculinity (27), our results show that depression and service use were retrospectively perceived as a resource to assist in changing harmful attitudes, e.g., toward internalized maladaptive work patterns. This is in line with recent systematic reviews of studies on the role of masculinity in men's mental health service use (4, 7). Qualitative studies have also explored the characteristics of positive attitudes during and after the mental help-seeking process (28, 29). Research has found that men developed positive coping strategies after utilizing mental health services by gaining a greater personal awareness during the recovery process. These strategies provided a new perspective on their situation, and they stopped striving for perfection in work and life (13). Alongside the hypothesis of a transformation of attitudes toward maladaptive work patterns among men with depression, our findings could also be discussed in the light of changing attitudes toward work in the general population. Results of a study about work values across generations suggests that workers from the generation of traditionalists placed more importance on status and autonomy than baby boomers or Generation X workers (30).

### Limitations and Future Research

Because study participants had used mental health care services prior to the study, results only refer to participants who had successfully sought help. Therefore, our findings are not able to explain reasoning processes in men who have never sought help for mental health problems. Another limitation is the small sample size as well as participants' high age (mean = 52), which means it is not possible to reveal age-related differences in help-seeking attitudes and behavior. However, there may be variances of dealing with depression in the light of society's expectations, e.g., relating to the male “breadwinner” role. Alongside the need for age-differentiated analyses, future studies could focus on fathers with depression to explore the meaning of fatherhood for coping with depression. While most of the reviewed literature demonstrated how masculine norms create barriers in seeking help for depression, more evidence on preferred types of service use is needed, e.g., in terms of the role of GPs as a potential point of contact for further information about mental health services. Furthermore, other findings suggest that the educational level is associated with the rigidity of gender roles, i.e., that a low level of education corresponds to rigid gender roles. Future research could focus on differences between milieus and along socio-demographic factors (i.e., education) to examine these hypotheses. Finally, our results show that it would be of great interest to conduct quantitative studies examining mental health needs among men with depression in a broader population. Although our study did not reveal any impact of socioeconomic status (SES) to masculinity orientations and service use behavior quantitative studies should include measurement of SES.

### Implications for Improving Help-Seeking Among Men With Depression

Despite its limitations, our study calls for interventions to improve help-seeking among men with depression. Findings highlight the need to consider perceived discrimination against men with depression. Interventions to reduce the stigma of being “unmanly” and to improve men's capacity to cope with being unable to work should be developed. Peer-led men-only groups may increase participants' self-esteem and assist in disclosing weaknesses. In the context of GPs' mediating role, training for health professionals concerning the impact of masculine norms on mental health is recommended. GPs competent in recognizing depressive symptoms may be able to play a key role in helping men by acting as a mediator for further psychiatric services. Finally, public campaigns are needed to change society's negative view of mental illnesses, help-seeking and service use among men as well as women with depression. One example approach that could be used to target the male population is the “*Real Men. Real Depression.”* campaign that aimed to increase public awareness and help other men recognize depression (31).

## Data Availability Statement

The original contributions presented in the study are included in the article/supplementary materials, further inquiries can be directed to the corresponding author/s.

## Ethics Statement

The studies involving human participants were reviewed and approved by the ethics committee of Ulm University, Germany (Ref. Nr. 202/15). The patients/participants provided their written informed consent to participate in this study.

## Author Contributions

SK, RK, HG, TB, and PB proposed the project idea. SK supervised the project. KF, MP, MSc, HG, and PB helped with participant recruitment. TS, AM-S, MSt, and SK undertook literature research and conducted and analyzed the interviews. TS drafted the manuscript. All authors contributed to and approved the final manuscript.

## Conflict of Interest

The authors declare that the research was conducted in the absence of any commercial or financial relationships that could be construed as a potential conflict of interest.
